# Establishment of noncycloplegic methods for screening myopia and pre-myopia in preschool children

**DOI:** 10.3389/fmed.2023.1291387

**Published:** 2023-12-14

**Authors:** Yao Yin, Liping Li, Ting Wang, Senlin Lin, Jia Wang, Hong Wang, Minmin Jiang, Yingyan Ma, Jianfeng Zhu

**Affiliations:** ^1^Department of Ophthalmology, Fengcheng Hospital, Fengxian District, Shanghai, China; ^2^Shanghai Eye Disease Prevention and Treatment Center, Shanghai Eye Hospital, Shanghai, China; ^3^Disease Control and Prevention Center in HongKou District, Shanghai, China; ^4^Department of Eye Disease Prevention and Treatment, Shanghai Yangpu District Kongjiang Hospital, Shanghai, China; ^5^Department of Ophthalmology, Shanghai General Hospital, Shanghai Jiao Tong University School of Medicine, Shanghai Key Laboratory of Fundus Diseases, National Clinical Research Center for Eye Diseases, Shanghai Engineering Center for Precise Diagnosis and Treatment of Eye Diseases, Shanghai, China

**Keywords:** myopia, preschoolers, pre-myopia, screening, accuracy

## Abstract

**Purpose:**

Pre-myopia, a non-myopic refractive state, is a key concern for myopia prevention because of its association with a significantly higher risk of myopia in children under 3 years of age. Amid the myopia pandemic, its onset at younger ages is increasing, yet research on screening methods for myopia and pre-myopia in preschool children remains limited. This study aimed to establish effective noncycloplegic screening methods for myopia and pre-myopia in preschool children.

**Methods:**

This cross-sectional study included 16 kindergartens in Shanghai, China. Uncorrected distance visual acuity (UDVA) was recorded using a logMAR visual acuity chart. Pre- and post-cycloplegic refractions were obtained using an auto-refractor (TopconKR-800). Noncycloplegic axial length (AL) and corneal curvature radius (CR) were measured using the IOL Master-700. Logistic regression models were developed to establish accurate noncycloplegic screening methods for myopia and pre-myopia.

**Results:**

A total of 1,308 children with a mean age of 4.3 ± 0.9 years were included; among them 640 (48.9%) were girls. The myopia prevalence rate was 2.8% (*n* = 36), and the prevalence of pre-myopia was 21.9% (*n* = 286). Pre-myopia screening (cycloplegic spherical equivalent [SE] ≤ −0.5 < SE ≤0.75 diopters [D]) using UDVA exhibited an area under the receiver operating curve (AUC) of 0.52, noncycloplegic SE had an AUC of 0.70 and AL had an AUC of 0.63. The accuracy of combining the SE and AL/CR ratio was among the best with the least number of checks used, and the AUC was 0.74 for pre-myopia screening and 0.94 for myopia screening (cycloplegic SE ≤ −0.5 D). The addition of UDVA did not further improve the accuracy.

**Conclusion:**

Using UDVA alone did not achieve good accuracy in pre-myopia or myopia screening of young children. Under non-cycloplegic conditions, the combination of AL/CR and SE demonstrated favorable results for pre-myopia and myopia screening of preschool children.

## 1 Introduction

Myopia has emerged as a significant global public health concern over the past decade. An estimated 30% of the world population was affected in 2020, this figure is expected to increase to 50% by 2050 (1). Prevalence is particularly high among East Asian adolescents, reaching up to 69% by age 15 years (2). Although Western countries experienced a modest increase in myopia prevalence, East Asian populations substantially increased by 23% (2). In 2019, a study of Chinese children and adolescents aged 7–18 years had an overall myopia prevalence of 60.1% (3). Given the irreversible complications associated with progressing to high myopia, retinal maculopathy and glaucoma, early intervention and prevention strategies are vital (4). Currently, interventions for myopia, including outdoor activities, atropine, progressive additional lens spectacles, bifocal spectacles, soft bifocal contact lenses, and orthokeratology, have demonstrated efficacy in slowing myopia progression (5). Therefore, early prevention, detection, and intervention are essential for controlling myopia in children (6).

In the past decade, the prevalence trend for myopia among preschoolers has also shifted, with peak onset now occurring at younger ages; decreasing from 12 years in 2010, 10 years in 2014, to 7 years in 2019 in Chinese children (3). Myopia prevalence in preschoolers in Shanghai is approximately 3%–4% (7, 8). Myopia has increased significantly among preschool children in Hong Kong, with the prevalence increasing from 2.3% in 1996 to 6.3% in 2006 (9). Similarly, the myopia prevalence among preschool children in Singapore is 6.1% (10). According to the International Myopia Research Institute, “pre-myopia” is a non-myopic refractive state, defined as a spherical equivalent (SE) between −0.50 diopters (D) and + 0.75 D, which, when combined with risk factors and observed eye growth patterns, is associated with a high risk of developing myopia; thus, preventive interventions are warranted (11). Pre-myopia prevalence in preschool children in Taiwan is as high as 52% (12). Despite this, research on myopia prediction largely focuses on school-aged children; thus, there is an urgent need to establish a screening model for myopia and pre-myopia in preschool-aged children.

Current screening methods for preschool children are primarily based on uncorrected distance visual acuity (UDVA). In a retrospective study of preschool children in New Zealand, UDVA was shown to provide rapid results, although it had a high rate of false-positive results and a low positive predictive value of 31% (95% confidence interval [CI]: 26 to 38%) (13). In some areas, screening for preschoolers is also conducted using a handheld vision screener. However, it was found that when performed under non-cycloplegia, handheld vision screener significantly underestimates refractive errors in preschoolers, with a low sensitivity (30%) for hyperopia (SE >3.5 D), as well as a low sensitivity (73.7%) for significant myopia (SE < −3.5 D) (14, 15). Thus, there is a distinct lack of methods for pre-myopia screening methods for preschool-aged children.

Cycloplegic refraction is the gold standard for the detection of refractive error in children (16). However, compliance with cycloplegic eye drops in preschool children is relatively poor, mainly due to the children’s resistance to eye drop administration, and parents’ fears regarding potential side effects (17). As a result, noncycloplegic refraction is commonly used in population-based epidemiologic studies of preschool children aged 3 to 6 years old (18, 19), although, this method can lead to an overestimation of myopia and pre-myopia prevalence (20). Constructing precise non-cycloplegic screening methods for preschoolers’ refractive status remains an important issue. In this cross-sectional study of preschoolers aged 3–6 years from the Shanghai Yangpu District, we investigated visual acuity, non-cycloplegic autorefraction and ocular biometrics (axial length [AL] and corneal curvature radius [CR]) to develop non-cycloplegic models for pre-myopia and myopia screening.

## 2 Methods

### 2.1 Study design

This cross-sectional study was conducted from October 2020 to January 2021 in 16 kindergartens randomly selected in Yangpu District, Shanghai, China. Preschool children aged 3–6 years from junior, middle and senior classes were included in the study. Children were excluded from the study if the consent to participate in the study was not obtained from children’s parents or their legal guardians. Children with systemic diseases such as congenital heart disease, ocular trauma or ophthalmic diseases such as glaucoma, cataract, and strabismus were also excluded. A total of 2,629 preschool children were included in the study. Among them, 1,309 parents or legal guardians of the children declined cycloplegic refraction examinations, and an additional 12 were excluded because they were unable to cooperate with the tests. Ultimately, 1,308 preschoolers who completed all the examinations were included in the analyses.

### 2.2 Examination procedures

The examination team included one ophthalmologist, six optometrists, and one public health physician. Before the study, all the members were trained and tested. All assessments for each child were completed in a single day and included visual acuity, intraocular pressure, autorefraction, and ocular biometrics. The results were recorded and uploaded to the online data acquisition system simultaneously.

UDVA measurements were collected using a standard logarithmic visual acuity chart. During the vision examination, the students stood 4 meters from the light box and the visual acuity of both eyes (first right, then left) was tested. Pre- and post-cycloplegic refraction were measured using an auto-refractor (KR-8800, Topcon, Tokyo, Japan). Refraction was measured three times, and the results were averaged. If any of the two measurements varied by more than 0.50 D, a further measurement was taken. Noncycloplegic AL and corneal curvature radius (CR) were measured using the IOL Master-700, which automatically took five measurements and calculated the average. All instruments were calibrated prior to examination. These procedures are well-established and detailed techniques are explained in our previous study (3).

For cycloplegia, the children were first anesthetized locally with one drop of 0.5% proparacaine hydrochloride in each eye. After approximately 15 s, 1 drop of 1% cyclopentolate hydrochloride eye drops (Alcon, Geneva, Switzerland) was placed in each eye twice at 5-min intervals. Pupil size and loss of light reflex were checked 30 min after the second drop, and cycloplegia was deemed complete if the pupil dilated to 6 mm or more and the light reflex disappeared. If the pupillary light reflex was still present, a third drop of cyclopentolate hydrochloride was placed in each eye. The researcher checked again after 15–20 min to see if the criteria for cycloplegia were met.

### 2.3 Statistical analyses

Due to the high correlation between the refractive results of both eyes, the right eye was chosen for data analysis. Logit models were used to develop joint screening methods. Several models were built using visual acuity, autorefraction, and ocular biometry to screen for myopia (cycloplegic SE ≤ −0.5 D) and pre-myopia (cycloplegic −0.5 D < SE ≤ +0.75 D). The SE was calculated as sphere power + 0.5*cylinder power.

Baseline characteristics were presented as counts (proportions) for categorical data and as mean ± standard deviation for continuous data. Chi-square tests were used to compare categorical variables. The distribution of all variables was examined for normality using the Kolmogorov–Smirnov test. For variables of normal distribution, Student’s t test was used for comparing differences between two groups and one-way analysis of variance was used for comparing differences between the three classes. For variables of nonnormal distribution, Wilcoxon’s rank sum test was applied. Receiver operating characteristic curves were constructed to obtain the best cut-off values, area under the curve (AUC), and Youden’s indices for each model. Sensitivity and specificity were also calculated for each model. Statistical analyses were performed using IBM SPSS Statistics (version 20.0; IBM Corp., Armonk, NY, United States) and SAS software (version 9.4; SAS Institute, Cary, NC, United States).

### 2.4 Ethical statements

The study adhered to the principles of the Declaration of Helsinki and was approved by the Ethics Committee of Shanghai General Hospital (2020SQ351). Written informed consent was obtained from the parents of all the participating children.

## 3 Results

The mean age of the 1,308 children included in the analyses was 4.3 ± 0.9 years; 668 boys (49.4%) and 640 girls (48.9%). Thirty-six (2.8%) and 286 (21.9%) children were diagnosed with myopia and pre-myopia, respectively. The number children in the junior classes (*n* = 425), middle classes (*n* = 421), and senior classes (*n* = 462) with myopia and pre-myopia were 6 (1.4%) and 91 (21.4%), (3.3%) and 87 (20.7%), and 16 (3.5%) and 108 (23.4%), respectively. Basic information about the children is displayed in Tables 1, 2. Although statistically significant differences were detected in age and visual acuity between the children included in the analyses and those who were excluded, the difference was not clinically valuable (Table 1). There were no significant differences in age or gender among the children with different refractive statuses (Table 2). However, significant differences were found in visual acuity, axial length, corneal refractive power, AL/CR ratio, and non-cycloplegic SE between the different refractive groups (Table 2).

**Table 1:** Basic information of entire children sample (*n* = 2,629).

	Children included in the analysis (*n* = 1,308)	Children excluded from the analysis (*n* = 1,321)	*p*-value
Age (mean ± SD)	4.27 ± 0.91	4.08 ± 0.92	<0.001^b^
Gender boy, no. (%)	668 (49.4%)	684 (50.6%)	0.72^a^
Girl, no. (%)	640 (50.1%)	637 (49.9%)	
Visual acuity (logMAR, mean ± SD)	0.24 ± 0.13	0.27 ± 0.13	<0.001^b^
Axial length (mean ± SD)	22.24 (0.73)		
Non-cycloplegic SE (mean ± SD)	0.14 ± 1.11	0.09 ± 1.29	0.30^b^
Cycloplegic SE (mean ± SD)	1.27 ± 0.94		

a Chi-square test.

b Student’s t test.

SD, standard deviation; SE, spherical equivalent.

**Table 2:** Basic information about the children included in the analysis (*n* = 1,308).

	Non-myopia (SE >0.75 D, *n* = 986)	Pre-myopia (−0.5 < SE ≤0.75 D, *n* = 286)	Myopia (SE ≤ −0.5 D, *n* = 36)	*p*-value
Age (mean ± SD)	4.26 ± 0.89	4.30 ± 0.97	4.53 ± 0.91	0.18^a^
Gender boy, no. (%)	493 (50.0%)	155 (54.2%)	20 (55.6%)	0.40^b^
Girl, no. (%)	493 (50.0%)	131 (45.8%)	16 (44.4%)	
Visual acuity (logMAR, mean ± SD)	0.24 ± 0.12	0.24 ± 0.13	0.41 ± 0.20	<0.001^c^
Axial length (mean ± SD)	22.13 ± 0.68	22.52 ± 0.73	23.32 ± 0.75	<0.001^c^
Corneal refractive power (mean ± SD)	43.47 ± 1.38	43.53 ± 1.59	43.511 ± 1.54	0.83^c^
AL/CR (mean ± SD)	2.87 ± 0.06	2.90 ± 0.06	3.00 ± 0.08	<0.001^c^
Non-cycloplegic SE (mean ± SD)	0.31 ± 1.00	−0.22 ± 1.01	−1.78 ± 1.81	<0.001^c^
Cycloplegic SE (mean ± SD)	1.61 ± 0.74	0.42 ± 0.30	−1.25 ± 1.09	<0.001^c^

a Analysis of variance.

b Chi-square test.

c Wilcoxon’s rank sum test.

AL, axial length; CR, corneal curvature radius; SD, standard deviation; SE, spherical equivalent.

Table 3 shows the screening accuracy of the different methods for myopia and pre-myopia using a single measurement. These results suggest that either AL/CR or non-cycloplegic SE alone is highly accurate for myopia screening (AUC > 0.9). For pre-myopia screening, the accuracy of either AL/CR or non-cycloplegic SE alone was moderate (0.7 < AUC ≤ 0.9).

**Table 3:** Summary for single-indicator screening accuracy.

	Screening target: myopia (SE ≤ −0.5 D)				Screening target: pre-myopia (−0.5 < SE ≤ 0.75 D)			
Measurement	Cut-off value^a^	Sensitivity^a^	Specificity^a^	AUC^b^	Cut-off value^a^	Sensitivity^a^	Specificity^a^	AUC^b^
AL/CR	2.93	0.86	0.86	0.92 (0.88 ~ 0.97)	2.86	0.76	0.58	0.71 (0.68 ~ 0.75)
Visual acuity	0.35	0.56	0.89	0.77 (0.69 ~ 0.86)	0.16	0.25	0.79	0.52 (0.48 ~ 0.55)
Non-cycloplegic SE	−0.31	0.97	0.83	0.93 (0.90 ~ 0.95)	0.44	0.86	0.48	0.70 (0.67 ~ 0.73)
AL	22.86	0.78	0.82	0.85 (0.78 ~ 0.92)	22.22	0.68	0.52	0.63 (0.60 ~ 0.67)

a Accuracy when the Youden’s index was maximized.

b Mean (95% confidence interval).

AL, axial length; AUC, area under the curve; CR, corneal curvature radius; SE, spherical equivalent refraction.

Table 4 lists the accuracy of the methods using multiple measurements. The results suggested that, for myopia screening, all the combined methods (model 1 to 4), were highly accurate (AUC > 0.9). For pre-myopia screening, the accuracy of the combined methods, which included AL/CR in the models (model 1, 2, and 4), was moderate (0.7 < AUC ≤ 0.9). However, the accuracy of the combined method without AL/CR (model 3) was low (AUC < 0.7). Notably, by combining AL/CR and non-cycloplegic SE (model 1) for pre-myopia screening, the AUC was comparable to that of combining all three methods (model 4).

**Table 4:** Accuracy of the combined screening model for myopia and pre-maturity^a^.

	Measurement			Myopia				Pre-myopia			
	AL/CR	Non-cycloplegic SE	Visual acuity	Sensitivity^b^	Specificity^b^	Youden Index	AUC^c^	Sensitivity^b^	Specificity^b^	Youden Index	AUC^c^
Model 1	**√**	**√**		0.81	0.96	0.77	0.94 (0.91 ~ 0.98)	0.71	0.65	0.36	0.74 (0.70 ~ 0.77)
Model 2	**√**		**√**	0.92	0.85	0.77	0.94 (0.91 ~ 0.98)	0.65	0.69	0.34	0.71 (0.68 ~ 0.74)
Model 3		**√**	**√**	0.86	0.77	0.63	0.90 (0.86 ~ 0.94)	0.74	0.41	0.15	0.58 (0.55 ~ 0.62)
Model 4	√	√	√	0.92	0.88	0.80	0.95 (0.92 ~ 0.99)	0.61	0.75	0.36	0.73 (0.70 ~ 0.77)

a Models were adjusted for age and sex.

b Accuracy when the Youden’s index was maximized.

c Mean (95% confidence interval).

AL, axial length; AUC, area under the curve; CR, corneal curvature radius; SE, spherical equivalent.

**Figure 1:**
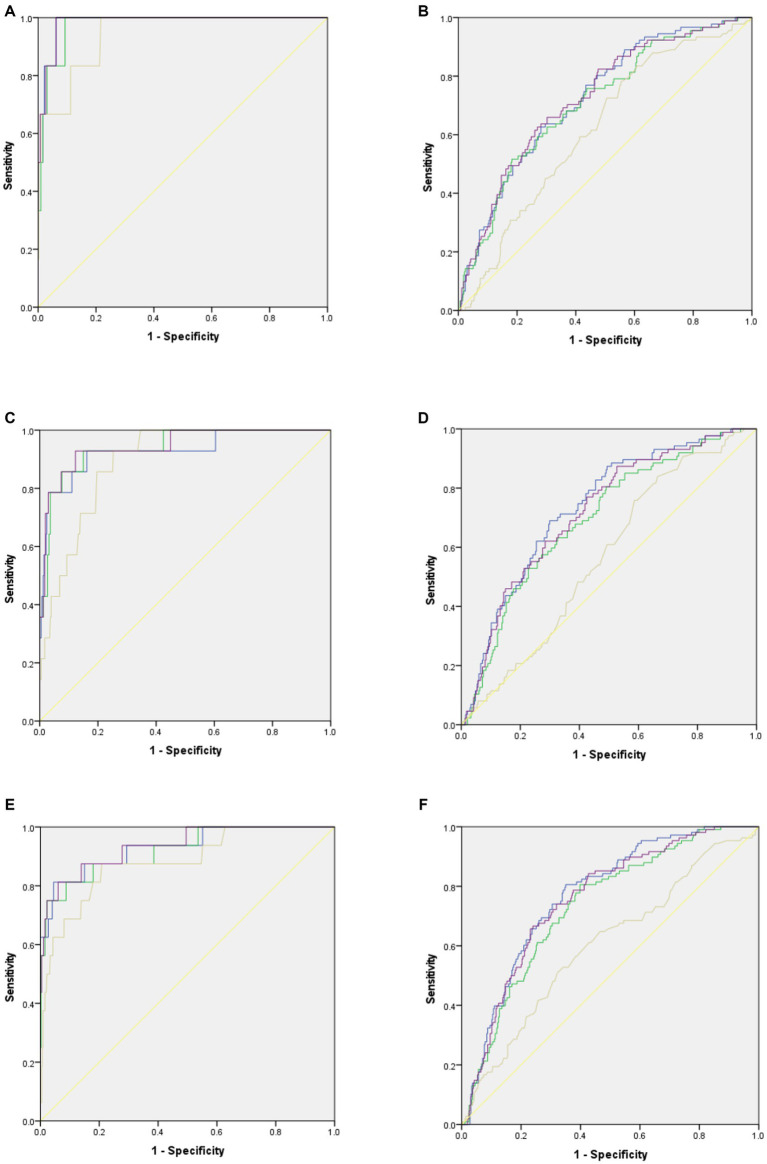
Accuracy of the combined screening models for myopia and pre-myopia screening in children of different grade levels. Blue curves stand for results of Model 1: genders, age, AL/CR, non-cycloplegic SE. Green curves stand for results of Model 2: genders, age, AL/CR, visual acuity. Brown curves stand for results of Model 3: genders, age, visual acuity, non-cycloplegic SE. Purple curves stand for results of Model 4: genders, age, visual acuity, non-cycloplegic SE, AL/CR. Yellow lines stand for the reference line. **(A)** ROC curves for myopia screening in junior classes. **(B)** ROC curves for pre-myopia screening in junior classes. **(C)** ROC curves for myopia screening in middle classes. **(D)** ROC curves for pre-myopia screening in middle classes. **(E)**: ROC curves for myopia screening in senior classes. **(F)**: ROC curves for pre-myopia screening in senior classes.

Figure 1 shows the ROC curves of the combination methods for myopia and pre-myopia screening for children of different classes. For screening myopia, the accuracy of model 1 was similar to that of model 4 for all three classes. The AUCs of model 1 and model 4 for myopia screening were 0.98 (95% CI: 0.96–1.00) and 0.98 (95% CI: 0.96–1.000) for the junior classes, 0.93 (95% CI: 0.85–1.00) and 0.94 (95% CI: 0.89–1.000) for the middle classes, and 0.93 (95% CI: 0.86–1.00) and 0.94 (95% CI: 0.87–1.00) for the senior classes, respectively.

For screening pre-myopia, the accuracy of model 1 was also comparable to that of model 4 for all three classes. The AUCs of model 1 and model 4 for pre-myopia screening were 0.73 (95% CI: 0.67–0.78) and 0.73 (95% CI: 0.67–0.78) for the junior classes, 0.74 (95% CI: 0.68–0.79) and 0.72(95% CI: 0.66–0.78) for the middle classes, respec and 0.77 (95% CI: 0.72–0.82) and 0.76 (95 CI: 0.71–0.81) for the senior classes, respectively.

## 4 Discussion

This study focused on the development of non-cycloplegic methods for myopia and pre-myopia screening for preschool-aged children. The results underscore the pivotal role of AL/CR measurements because the accuracy of the screening methods without AL/CR was low.

The prevalence of myopia in young children is increasing annually. Shanghai, in particular, exhibits one of the highest myopia detection rates in China (21). Due to the rapid development of the economy, an increasing number of preschool children are being exposed to digital screens (e.g., smartphones) at an early age, which increases the risk of myopia (22). Although visual acuity charts have been the go-to tools for myopia screening, their diagnostic accuracy for significant refractive errors is relatively high (e.g., for hyperopia >3.25 D, myopia < −2.00 D, and astigmatism >1.50 D). Since the preschool children’s cooperation is limited during these examinations, the reliability of the screening results may have been compromised. Additionally, because visual acuity may not decrease in early myopia cases, relying on visual acuity alone becomes challenging (23–25). In our study, the sensitivities of visual acuity alone for screening myopia and pre-myopia were 56% and 25%, respectively. Therefore, we suggest that visual acuity alone is insufficient for myopia and pre-myopia screening in preschool children (25).

Auto-refractors have been frequently used in myopia screening programs for preschool children (14); however, non-cycloplegic refraction specificity remains low owing to strong accommodation, especially in preschool children (16). Our study revealed that combining AL/CR and non-cycloplegic refraction (model 1) yielded accurate results for myopia screening of preschool children, with a sensitivity and specificity of 81% and 94%, respectively. AL/CR has been suggested to be more accurate than AL alone for defining refraction in both school-aged and preschool children, supporting the use of AL/CR in screening strategies (26, 27). In general, adding more tests could increase screening accuracy, however, model 1 had similar accuracy for myopia and pre-myopia screening compared with the combination of all three tests (model 4). Therefore, from the perspective of practical application, model 1 is recommended because it uses fewer examinations and can save human resources and equipment costs.

However, this combined method (model 1) displayed relatively lower sensitivity and specificity for pre-myopia screening (71% and 65%, respectively). This decrease in accuracy can be attributed to the larger discrepancy between non-cycloplegic and cycloplegic autorefraction in the emmetropic and hyperopic eyes, especially in preschool children (20). Although adding AL/CR improved the accuracy of pre-myopia screening, it still did not achieve satisfactory results compared with the accuracy of myopia screening, potentially due to the different patterns of AL and CR changes in the pre-myopic status of preschool children, who are still undergoing refractive development.

Notably, pre-myopia exhibited a high prevalence among preschool children (12). Children with pre-myopia may have an increased risk of developing myopia in the future. Thus, timely health promotion and regular follow-up are crucial for reducing the risk of myopia. Although, adjusting cut-off values of the combined method could maximize the Youden’s index for pre-myopia screening, a higher cut-off value with higher sensitivity is recommended.

Our study had several limitations. First, the high cost of the IOL Master-700 may limit the availability of using the combined AL/CR and non-cycloplegic refraction method in economically disadvantaged areas. Additionally, our study was conducted only in Shanghai, and the distribution of refractive status in preschool children may differ among different areas. Therefore, if applied to other areas, the screening method parameters and cut-off values should be re-evaluated.

## 5 Conclusion

This study offers a systematic analysis of various methods for myopia and pre-myopia screening of preschool children, and emphasizes the value of AL/CR measurements. Our study suggests that using uncorrected visual acuity alone does not achieve good accuracy; the AL/CR measurement was more valuable in pre-myopia or myopia screening of young children. Under non-cycloplegic conditions, the combination of AL/CR and SE may provide favorable results for pre-myopia and myopia screening of preschool-aged children.

## Data availability statement

The raw data supporting the conclusions of this article will be made available by the authors, without undue reservation.

## Ethics statement

The studies involving humans were approved by Ethics Committee of Shanghai General Hospital (2020SQ351). The studies were conducted in accordance with the local legislation and institutional requirements. Written informed consent for participation in this study was provided by the participants’ legal guardians/next of kin.

## Author contributions

YY: Writing – original draft, Conceptualization, Investigation, Methodology, Visualization. LL: Writing – original draft, Conceptualization, Methodology, Visualization. TW: Investigation, Writing – review & editing. SL: Methodology, Supervision, Writing – review & editing. JW: Investigation, Writing – review & editing. HW: Resources, Supervision, Writing – review & editing. MJ: Conceptualization, Resources, Supervision, Writing – review & editing. YM: Conceptualization, Methodology, Supervision, Writing – review & editing. JZ: Conceptualization, Project administration, Resources, Supervision, Writing – review & editing.
