# Corrigendum: Establishment of noncycloplegic methods for screening myopia and pre-myopia in preschool children

**DOI:** 10.3389/fmed.2024.1414287

**Published:** 2024-04-30

**Authors:** Yao Yin, Liping Li, Ting Wang, Senlin Lin, Jia Wang, Hong Wang, Minmin Jiang, Yingyan Ma, Jianfeng Zhu

**Affiliations:** ^1^Department of Ophthalmology, Fengcheng Hospital, Fengxian District, Shanghai, China; ^2^Shanghai Eye Disease Prevention and Treatment Center, Shanghai Eye Hospital, Shanghai, China; ^3^Disease Control and Prevention Center in HongKou District, Shanghai, China; ^4^Department of Eye Disease Prevention and Treatment, Shanghai Yangpu District Kongjiang Hospital, Shanghai, China; ^5^Department of Ophthalmology, Shanghai General Hospital, Shanghai Jiao Tong University School of Medicine, Shanghai Key Laboratory of Fundus Diseases, National Clinical Research Center for Eye Diseases, Shanghai Engineering Center for Precise Diagnosis and Treatment of Eye Diseases, Shanghai, China

**Keywords:** myopia, preschoolers, pre-myopia, screening, accuracy

In the published article, there was an error in the Funding statement. The funding number of the Shanghai Three-year Action Plan for Strengthening the Construction of Public Health System (2023−2025) Youth Project was incorrectly written as:“GWVI-11.2-YQ6”. The correct Funding statement appears below.

